# Supplementary material for: Role of Operating Conditions in a Pilot Scale Investigation of Hollow Fiber Forward Osmosis Membrane Modules
Source: Membranes (Basel). 2019 Jun 3;9(6):66. doi: 10.3390/membranes9060066 (PMC6631378; doi:10.3390/membranes9060066)
Supplement: Supplementary file 1 [file membranes-09-00066-s001.pdf]

membranes

Supplementary Materials: Role of Operating Conditions in a Pilot Scale Investigation of Hollow Fiber Forward Osmosis Membrane Modules

Victoria Sanahuja-Embuena [1,2,*], Gabriel Khensir [3], Mohamed Yusuf [3], Mads Friis Andersen [2], Xuan Tung Nguyen [2], Krzysztof Trzaskus [2], Manuel Pinelo [3], Claus Helix-Nielsen [1,*]

1 Department of Environmental Engineering, Technical University of Denmark, Miløvej 113, 2800 Kongens Lyngby, Denmark
2 Aquaporin A/S, Nymøllevej 78, 2800 Kongens Lyngby, Denmark; mfr@aquaporin.com (M.F.A.); xtn@aquaporin.com (X.T.N.); ktr@aquaporin.com (K.T.)
3 Department of Chemical and Biochemical Engineering, Technical University of Denmark, Søltofts Plads, 2800 Kongens Lyngby, Denmark; gabiih@live.dk (G.K.); Moe0207@hotmail.com (M.Y.)
* Correspondence: vics@env.dtu.dk (V.S.-E.); clhe@env.dtu.dk (C.H.-N.); Tel.: +45-53-555-503 (V.S.-E.); Tel.: +45-42-550-750 (C.H.-N.)

Received: 29 April 2019; Accepted: date; Published: date

S1. Specifications of Aquaporin Inside™ HFFO220 Module

Detailed information of the commercial HFFO module used in this study can be found in Table S1.

Table S1. Specifications of the Aquaporin Inside™ HFFO2 module used in all the tests. ID, inner diameter; OD, outer diameter.

Aquaporin Inside™ HFFO2 module				
**Membrane Area**	**Fiber ID**	**Fiber OD**	**Number of Fibers**	**Fiber Length**
**m²**	**mm**	**mm**	**-**	**mm**
2.30	0.20	0.27	30800	270

S2. Evaluation of the Water Permeability Coefficient A, Solute Permeability Coefficient B and Structural Parameter S for HFFO Modules

To evaluate the intrinsic characteristics of HFFO membranes, it is essential to understand the forward osmosis process and its governing mechanisms. Figure S1 shows a schematic of the solute concentration gradients—Including ICP and ECP—across a thin-film composite membrane operating in FO mode (active-layer facing FS). The effective driving force for forward osmosis exists only between the interfaces of the selective polyamide layer. From the bulk DS, solutes must diffuse through the porous support to the interface between the support and selective layer. Once there, a small amount of solute diffuses across the selective layer to the lower-osmotic pressure FS. In addition, water molecules, moving from feed to draw, dilute the solute concentration on the porous substrate surface, creating a reduction in concentration between draw bulk solution and support layer surface (dilutive ECP). Conversely, water extraction from feed to draw, coupled with diffusion from solute across the selective layer, creates a zone where solute concentration is higher than in the feed bulk solution (concentrative ECP).

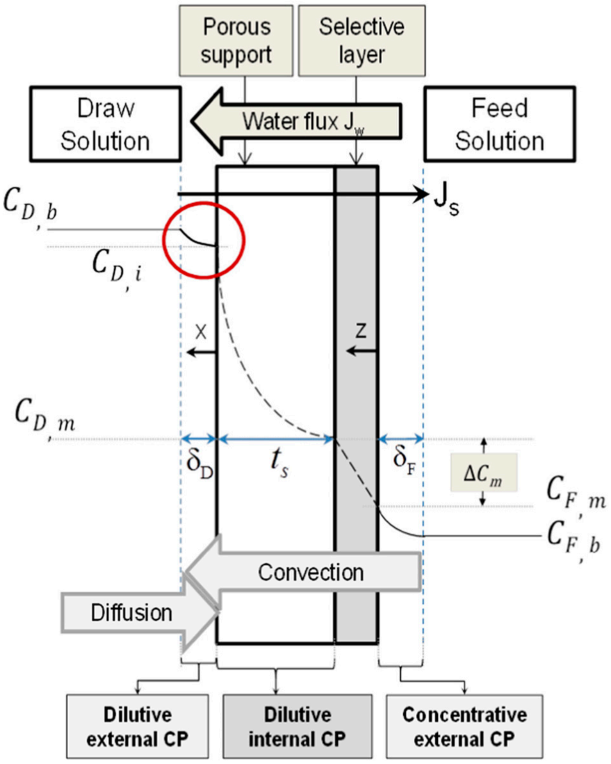

Figure S1. Illustration of the solute concentration profile at steady state across a thin film composite membrane in FO mode (active-layer facing FS), Adapted from Bui et al. [1].

These solution concentration gradients are mainly governed by intrinsic membrane parameters, namely A in $L{\cdot}m^{-2}{\cdot}h^{-1}{\cdot}bar^{-1}$, B in $L{\cdot}m^{-2}{\cdot}h^{-1}$, and S parameter in µm. The S parameter can be described as the membrane path length for the diffusion of solutes and is calculated based on the tortuosity, porosity and thickness of the membrane support structure. Hydrodynamic conditions such as cross-flow velocities, viscosities, densities of FS and DS, and diffusivities of draw solutes directly impact mass transfer coefficients of the solutes at the different regions across the thin film composite membrane. For evaluation of the intrinsic parameters of the membrane (A, B, and S parameters), a model developed by Bui et al. [1] accounting for ICP, dilutive and concentrative ECP, coupled with the J_s results is utilized. In this model, J_w and J_s are calculated according to Equations S1 and S2, respectively:

$$J_w = A\left\{\frac{\pi_{D,b}\exp\left[-J_w\left(\frac{1}{k_D}+\frac{S}{D_D}\right)\right] - \pi_{F,b}\exp\left(\frac{J_w}{k_F}\right)}{1 + \frac{B}{J_w}\left\{\exp\left[\frac{J_w}{k_F}\right] - \exp\left[-J_w\left(\frac{1}{k_D}+\frac{S}{D_D}\right)\right]\right\}}\right\} \quad \text{(S1)}$$

$$J_s = B\left\{\frac{c_{D,b}\exp\left[-J_w\left(\frac{1}{k_D}+\frac{S}{D_D}\right)\right] - c_{F,b}\exp\left(\frac{J_w}{k_F}\right)}{1 + \frac{B}{J_w}\left\{\exp\left[\frac{J_w}{k_F}\right] - \exp\left[-J_w\left(\frac{1}{k_D}+\frac{S}{D_D}\right)\right]\right\}}\right\} \quad \text{(S2)}$$

where π is the osmotic pressure of the DS, c is the solute concentration, k is the mass transfer coefficient, and D is the solute diffusivity.

While many papers have evaluated the A, B, and S parameters for flat sheet coupons or lab scale hollow fiber modules using a similar method, this is the first instance where the A, B, and S parameters are evaluated for an industrial HFFO module with a significantly larger surface area. The resulting large permeation through the membrane causes a significant change of the hydrodynamic conditions between the inlet and outlet of the module. For instance, the outlet feed stream has a significantly lower crossflow velocity, and a higher solute concentration than inlet feed stream. Therefore, another layer of non-linearity is built upon the Bui et al. [1] model to express π, c, k, and D as average parameters between inlet and outlet, and their respective dependency on J_w and J_s. For

instance, for feed and draw side, an average crossflow velocity ($\bar{v}_F$ and $\bar{v}_F$) is defined according to Equations S3 and S4, respectively:

$$\bar{v}_F = \frac{1}{2} \cdot \left[v_{F,in} + \frac{Q_{F,in} - J_w \cdot S_A}{S_{CSF}} \right] \quad (S3)$$

$$\bar{v}_D = \frac{1}{2} \cdot \left[v_{D,in} + \frac{Q_{D,in} + J_w \cdot S_A}{S_{CSD}} \right] \quad (S4)$$

where $v_{F,in}$ and $v_{D,in}$ are feed and draw cross-flow velocity at the inlet to the module, $Q_{F,in}$ and $Q_{D,in}$ are volumetric flows rated for feed and draw at the inlet to the module, S_A is the surface area of the module and S_{CSF} and S_{CSD} are cross-sectional areas for feed and draw sides, respectively. The same logic is applied for calculation of the average solute concentration on the feed and draw side:

$$\bar{c}_F = \frac{1}{2} \cdot \left[c_{F,in} + \frac{Q_{F,in} \cdot c_{F,in} + J_s \cdot S_A}{Q_{F,in} - J_w \cdot S_A} \right] \quad (S5)$$

$$\bar{c}_D = \frac{1}{2} \cdot \left[c_{D,in} + \frac{Q_{D,in} \cdot c_{D,in} - J_s \cdot S_A}{Q_{D,in} + J_w \cdot S_A} \right] \quad (S6)$$

where $c_{F,in}$ and $c_{D,in}$ are feed and draw solute concentrations at the inlet to the module. In the same way an average density $\bar{\rho}_{F(D)}$, viscosity $\bar{\eta}_{F(D)}$ and diffusivity $\bar{D}_{F(D)}$ are estimated for the feed and draw streams:

$$\bar{\rho}_{F(D)} = f(\bar{c}_{F(D)}) \ ; \ \bar{\eta}_{F(D)} = g(\bar{c}_{F(D)}) \ ; \ \bar{D}_{F(D)} = h(\bar{c}_{F(D)}). \quad (S7)$$

Function f(c), g(c), and h(c) are empirical correlations [2] that express dependence of the solute concentration on density, viscosity and diffusivity at ambient temperature, respectively. An average Schmidt number for FS and DS, $\overline{Sc}_{F(D)}$, can be, therefore, calculated according to Equation S8:

$$\overline{Sc}_{F(D)} = \frac{\bar{\eta}_{F(D)}}{\bar{D}_{F(D)} \cdot \bar{\rho}_{F(D)}} \quad (S8)$$

An average Reynolds number $\overline{Re}_{F(D)}$ for FS and DS is calculated from Equation S9:

$$\overline{Re}_{F(D)} = \frac{\bar{v}_{F(D)} \cdot d_{h,D(F)} \cdot \bar{\rho}_{F(D)}}{\bar{\eta}_{F(D)}} \quad (S9)$$

where $d_{h,i}$ represents hydraulic diameters for the feed or for draw side. For the HFFO modules, $d_{h,F}$ is just fiber inner diameter (ID = 195 μm) and $d_{h,D}$ is calculated from Equation S10 to be 226 μm for the shell side.

$$D_{h,D} = \frac{4 \cdot S_{CSD}}{C_w} \quad (S10)$$

where C_w is the internal area of the cross-section. For the feed side, to assess the Sherwood number (Sh), the Graetz number (Gz) can be calculated according to the Shen et al. [2] correlation:

$$\overline{Gz}_F = \frac{\overline{Re}_F \cdot d_{h,F} \cdot \overline{Sc}_F}{l} \quad (S11)$$

where l indicates the length of the fiber in HFFO modules, which is 275 mm. The Sherwood number (Sh) for the feed side was calculated from Equation S12 and S13:

$$\overline{Sh}_F = 1.62 \times \overline{Gz}_F^{\,0.33} \quad \text{for} \quad \overline{Gz}_F \geq 6 \quad (S12)$$

$$\overline{Sh}_F = 0.50 \times \overline{Gz}_F^{\,1.0} \quad \text{for} \quad \overline{Gz}_F < 6. \quad (S13)$$

For the shell-side correlation proposed by Asimakopoulou et al. [3] for a liquid-liquid system with a fiber dimension similar to those in the HFFO modules (ID ~ 200 μm, and packing ~ 50%) the following equation was selected:

$$\overline{Sh}_D = 1.615 \cdot (0.6 + 1.7\emptyset) \cdot \left[\frac{\overline{Re}_D \cdot d_{out} \cdot \overline{Sc}_D}{l}\right]^{0.33} \text{ for } 3 < Re < 75 \text{ and } 0.05 < \emptyset < 0.45 \quad (S14)$$

where d_{out} is the outer dimeter of fiber (265 μm) and $\emptyset$ is the packing density of the hollow fiber module. Other unknowns in Equations S1 and S2 such as π, c, k, D, J_w, and J_s can be experimentally determined, leaving behind only three unknowns (A, B, and S parameters) to be simultaneously solved from this system of non-linear equations. At each experimental condition of pre-selected feed and draw solute concentrations, two equations can be set-up for J_w and J_s respectively. In order to determine the A, B and S parameters for a particular solute type, four sets of experimental conditions with various draw solution concentrations were conducted, establishing, in total, 8 sets of equations, where the A, B, and S parameters are the only unknowns. With 8 equations and 3 unknowns, we have an over-specified system of equations, which are usually redundant but necessary in this case to account for possible experimental errors. Therefore, an RMSE minimization problem was set-up:

$$\text{minRMSE} = \min\left(\sqrt{\frac{\sum_1^n (J_{w,exp_n} - J_{w,predicted_n})^2 + \sum_1^n (J_{s,exp_n} - J_{s,predicted_n})^2}{2n}}\right) \quad (S15)$$

where n is the number of experiments sets, each with distinct bulk concentration of DS and corresponding J_w and J_s. Lastly, Matlab code was compiled to evaluate the intrinsic parameters the A, B, and S parameters based on the model developed above. Matlab optimization function selects the A, B, and S parameters to predict J_w and J_s based on Bui et al. [1] model. The A, B, and S parameters are determined when RMSE errors are minimized. In addition, R^2 for J_w and J_s are maximized to achieve best fitting of experimental and theoretical values. Constraints set for the A, B, and S parameters are as follow: 0.5 $L \cdot m^{-2} \cdot h^{-1} \cdot bar^{-1} < A < 6.0\ L \cdot m^{-2} \cdot h^{-1} \cdot bar^{-1}$, 0.01 $L \cdot m^{-2} \cdot h^{-1} < B < 2.00\ L \cdot m^{-2} \cdot h^{-1}$, 0.03 mm < S parameter < 0.50 mm. Minimum value of the S parameter is set based on the known wall-thickness of the hollow fiber, while the range of A and B values are set based on the LPRO results that determines water permeability and NaCl solute rejection for the tested modules. The model may yield solutions with a minimum RMSE, but that greatly differs from the experimental values of A and B. Consequently, some qualitative assessment of solutions of the minimization function was needed to evaluate these intrinsic parameters. Some constraint values for the model were changed to allow for more realistic and reasonable results, which are closer to experimental values of the A and B. The testing conditions, setup design, and governing equations used to carry out experimental evaluation of the A, B, and S parameters are described in section 2.2.3.

S3. Statistical Analysis

The statistical software JMP Home (SAS) was used to analyze the compared data in this study. Depending on the sample size of each experiment, the software recommended the corresponding type of ANOVA (one-way, two-way) to calculate the probability values (p-value) of significance. The probability value used as reference was 0.05, where anything below that number represents a significant difference between factors. Table S2 shows the p-values of the compared J_w between the two types of HFFO modules and between the variation of each test condition. In contrast, Table S3 shows the p-values of the compared J_s/J_w values. For example, in Table S2, for the test where feed flow rate was varied from 60 to 140 Lh^{-1}, the p-value shows that there is a significant difference in J_w between different flow rates for both membranes. Furthermore, the p-value for the membranes shows that there is a significant difference between the HF-C and HF-O modules in terms of J_w.

Table S2. Probability values (p-value) from the statistical analysis ANOVA of J_w between the two types of HFFO (HF-C, HF-O) and between the tests studied in this article. "Membranes" represents the significant differences in J_w between HF-C and HF-O. "Test" represents the significant differences in J_w for each test conditions for both HF-C and HF-O. p-values < 0.05 show a significant difference between the groups.

ANOVA Analysis - Response Factor Analysed: J_w			
**FS: RO water**	**p-value < 0.05**	**FS: Artificial Seawater**	**p-value < 0.05**

Feed flow rate	Membranes	✓	Feed flow rate	Membranes	✗
	Test	✓		Test	✓
Draw flow rate	Membranes	✓	Draw flow rate	Membranes	✗
	Test	✓		Test	✓
TMP	Membranes	✓	TMP	Membranes	✗
	Test	✗		Test	✗
PRO vs FO	Membranes	✓	PRO vs FO	Membranes	✗
	Test	✗		Test	✗
Flow orientation	Membranes	✗	Flow orientation	Membranes	✗
	Test	✗		Test	✗
Temperature	Membranes	✓			
	Test	✓			
**Draw Solute Tests**		**p-value < 0.05**			
NaCl	Test	✓			
	Membranes	✗			
$MgCl_2$	Test	✓			
	Membranes	✓			
$MgSO_4$	Test	✓			
	Membranes	✓			

Table S3. Probability values (p-value) from the statistical analysis ANOVA of J_s/J_w between the two types of HFFO (HF-C and HF-O) and between the tests studied in this article. "Membranes" represents the significant differences in J_s/J_w between HF-C and HF-O. "Test" represents the significant differences in J_s/J_w for each test condition for both HF-C and HF-O. p-values < 0.05 show a significant difference between the groups.

**ANOVA Analysis - Response Factor Analysed: J_s/J_w**					
**FS: RO Water**		**p-value < 0.05**	**Draw Solute Tests**		**p-value < 0.05**
Feed flow rate	Membranes	✓	NaCl	Membranes	✓
	Test	✗		Test	✗
Draw flow rate	Membranes	✓	$MgCl_2$	Membranes	✓
	Test	✗		Test	✗
TMP	Membranes	✓	$MgSO_4$	Membranes	✓
	Test	✗		Test	✗
PRO vs FO	Membranes	✓			
	Test	✓			
Flow orientation	Membranes	✓			
	Test	✗			
Temperature	Membranes	✓			
	Test	✗			

References

1. Bui, N.N.; Arena, J.T.; McCutcheon, J.R. Proper accounting of mass transfer resistances in forward osmosis: Improving the accuracy of model predictions of structural parameter. *J. Memb. Sci.* **2015**, *492*, 289–302.
2. Shen, S.; Kentish, S.E.; Stevens, G.W. Shell-side mass-transfer performance in hollow-fiber membrane contactors. *Solvent Extr. Ion Exch.* **2010**, *28*, 817–844.
3. Asimakopoulou, A.G.; Karabelas, A.J. A study of mass transfer in hollow-fiber membrane contactors-The effect of fiber packing fraction. *J. Memb. Sci.* **2006**, *282*, 430–441.
